# Short-Rotation Coppice Pellets as Biofuels in a Modern Boiler with an Electrostatic Precipitator: Particulate Matter and Carbon Monoxide Emissions

**DOI:** 10.3390/molecules31193443

**Published:** 2026-09-27

**Authors:** Kazimierz Warmiński, Paweł Dudziec, Adam Nocoń, Mariusz Jerzy Stolarski

**Affiliations:** 1 Department of Chemistry, Faculty of Agriculture and Forestry, University of Warmia and Mazury in Olsztyn, 10-719 Olsztyn, Poland; 2 Centre for Bioeconomy and Renewable Energies, University of Warmia and Mazury in Olsztyn, 10-727 Olsztyn, Poland; mariusz.stolarski@uwm.edu.pl; 3 Department of Plant Breeding and Bioresource Engineering, Faculty of Agriculture and Forestry, University of Warmia and Mazury in Olsztyn, 10-724 Olsztyn, Poland; pawel.dudziec@uwm.edu.pl; 4 Chamber of Commerce for Renewable Energy Devices, 41-250 Czeladź, Poland; kontakt@adamnocon.pl

**Keywords:** willow, poplar, black locust, particulate matter, carbon monoxide, combustion efficiency, woody biomass, small-scale boiler, biomass combustion, emission factors

## Abstract

Alternative pellet feedstocks must be evaluated for energy performance and air pollutant emissions. This study assessed particulate matter (PM) and carbon monoxide (CO) emissions from short-rotation coppice (SRC) pellets combusted in a pellet boiler equipped with an electrostatic precipitator (ESP). Pine and birch pellets served as references. Emissions were measured with and without the ESP and expressed per flue gas volume, fuel energy, and fuel mass. Pellet species significantly affected PM and CO, whereas the ESP significantly reduced PM but did not significantly affect CO. The ESP reduced mean PM concentrations by 52.5%, from 53.81 to 25.54 mg m^−3^. The highest PM concentration occurred for willow without the ESP (90.96 mg m^−3^), and the lowest for pine with the ESP (17.51 mg m^−3^); the highest value was 419.5% greater. Poplar produced relatively low PM but the highest CO emissions. Among SRC fuels, black locust provided the most balanced profile, combining relatively low PM and CO with the highest combustion efficiency (93.20%). Pine and birch, particularly with the ESP, produced the lowest emissions. These findings indicate that SRC pellets showed emission suitability for use in the tested boiler based on PM and CO emission levels.

## 1. Introduction

Climate change and the need to reduce fossil fuel consumption have increased the importance of renewable energy sources, including solid biomass used for heat generation. Biomass is considered an accessible and storable energy carrier, and its combustion is frequently treated as nearly carbon-neutral because the carbon dioxide released during burning had previously been absorbed by plants during growth (caveat: carbon neutrality depending, among other things, on cultivation, logistics, land-use change, processing, and the time horizon) [1,2]. At the same time, this fuel cannot be regarded as environmentally neutral in every respect. Biomass combustion releases particulate matter and gaseous pollutants, and the final emission profile depends strongly on the fuel properties, combustion technology and the presence or absence of flue-gas cleaning systems [3,4]. Particulate matter emitted during biomass combustion is a complex mixture of coarse ash, fine inorganic aerosols, soot, unburned carbon, and condensable organic compounds [5,6]. Fine particles may form through the volatilization and condensation of ash-forming elements, particularly potassium, sodium, chlorine, and sulfur, whereas soot and organic particles are mainly associated with incomplete oxidation. Consequently, PM emissions depend strongly on fuel ash composition, moisture content, pellet quality, combustion temperature, air distribution, and fuel-feeding stability [6,7]. Fuels rich in alkali metals and chlorine may therefore generate high inorganic PM emissions even when combustion is relatively complete. Carbon monoxide is an intermediate product of carbon oxidation and a widely used indicator of incomplete combustion. Its emissions increase under insufficient temperature, poor fuel–air mixing, uneven oxygen supply, or inadequate gas residence time [8,9]. However, PM and CO emissions are not always directly correlated: unfavorable combustion may increase both CO and carbonaceous PM, whereas high inorganic PM may also occur at relatively low CO levels [5].

Pellets are among the most convenient solid biomass fuels for small-scale and domestic heating. Compared with uncompacted biomass, they are characterized by lower moisture, higher density, uniform shape, easier transport and storage, and more stable combustion conditions [10,11]. Wood pellets are widely used in Europe and are usually produced from forest dendromass and wood-processing residues, including pine and birch, which generally provide favorable fuel properties, high lower heating value (LHV) and low ash content [12]. However, the increased price of high-quality wood pellets, intensified by the recent energy crisis, has encouraged the search for cheaper and locally available alternatives, including agro-biomass and biomass from dedicated perennial crops [13].

Short-rotation coppice (SRC) crops, such as willow, poplar and black locust, are an important group of potential feedstocks for pellet production. Their multiannual cultivation, regrowth after harvest, relatively low nutrient demand and limited agrotechnical requirements may reduce greenhouse gas emissions and increase carbon accumulation in soil [14]. Fast-growing woody species cultivated in short rotation cycles are therefore increasingly considered as dedicated energy crops capable of producing lignocellulosic biomass while contributing to carbon sequestration [15]. Recent European-scale data indicate that short-rotation and other fast-growing biomass production systems are already established across a considerable area. Pineda-Zapata and Mola-Yudego [16] documented 426,783 fields and stands of biomass production systems across 17 European countries and regions, covering 2,140,568 ha. Poplar occupied 341,164 ha, black locust 46,844 ha, and willow 16,282 ha. Poplar showed the widest spatial distribution, whereas willow was concentrated mainly in northern Europe, particularly Denmark and Sweden. The authors also emphasized that data on plantation systems remain fragmented and insufficiently standardized across Europe, which limits comprehensive assessment of biomass availability and cultivated area. Although SRC biomass may be heterogeneous in its primary form, pelletization can improve its usability and make it suitable for local heating plants as well as small individual boilers [17,18]. The pellet production process, based on drying, grinding and extrusion under high pressure and temperature, enables the transformation of SRC biomass into a standardized cylindrical fuel [19].

The wider use of biomass in domestic heating is limited by emissions of particulate matter and carbon monoxide. Residential biomass combustion has been identified as one of the major sources of atmospheric pollution in Europe [20,21]. At the global scale, the HTAP_v3 inventory indicates that residential combustion accounted for approximately one quarter of anthropogenic PM_2.5_ emissions and about one third of anthropogenic CO emissions in 2018 [22]. However, global inventories generally combine biomass with coal and other household fuels; therefore, these values should not be interpreted as biomass-only contributions. In the EU-27, heating and cooling activities generated 73% of total PM_2.5_ and 61% of total CO emissions in 2022, of which residential sources accounted for 85% and 76%, respectively [23]. This corresponds to approximately 62% of total PM_2.5_ and 46% of total CO emissions, although the estimate includes both biomass and non-biomass residential fuels. Small combustion installations are particularly relevant because they are often located close to people and emit pollutants through low stacks. In such conditions, particulate matter contributes to winter smog episodes, especially in densely populated areas and poorly ventilated regions such as valleys [24]. PM exposure is associated with cardiovascular and respiratory diseases, while the smallest particles can penetrate deep into the respiratory tract and, in the case of ultrafine particles, even reach the circulatory system [25,26]. In addition to particle size, the health effects of particulate matter depend on its chemical composition and oxidative potential. Combustion-derived PM may contain transition metals, polycyclic aromatic hydrocarbons, black carbon, and other compounds capable of generating reactive oxygen species. These processes can induce oxidative stress, airway inflammation, endothelial dysfunction, and systemic inflammatory responses [27]. Recent epidemiological evidence also indicates that exposure to specific PM_2.5_ components may aggravate cardiovascular disease and increase the risk of adverse outcomes, including in-hospital case fatality among patients with acute myocardial infarction [28]. Consequently, PM mass concentration alone may not fully reflect the health risk associated with biomass combustion emissions. Carbon monoxide represents an additional health hazard because it is colorless and odorless and reduces oxygen delivery to tissues through the formation of carboxyhemoglobin. Exposure may cause headache, dizziness, confusion, cardiovascular stress, neurological injury, loss of consciousness, and death [29,30].

The emission risk from biomass combustion is not determined only by the mass of emitted dust. Particles may contain ash-forming minerals, heavy metals, unburned carbon and polycyclic aromatic hydrocarbons, and their toxicity depends on chemical composition, physical state and size distribution [31,32]. The properties of emitted fly ash and dust vary with the type of wood or biomass fuel. Moreover, the operation and construction of the heat source—combustion chamber, burner, air distribution, heat exchanger and flue-gas path—strongly influence combustion efficiency and emission formation [33]. Therefore, matching the correct fuel with an appropriate technology and emission control system is necessary to obtain high efficiency and low pollutant emissions [34].

Primary measures, such as improved boiler design, air-flow regulation and fuel-oriented approaches, can reduce pollutant formation, but they are often insufficient for meeting stricter emission requirements [35]. For this reason, secondary flue-gas cleaning methods are increasingly important. Among them, electrostatic precipitators (ESPs) are considered particularly promising for small-scale biomass combustion because they can remove fine and ultrafine particles with relatively low pressure drop, low energy consumption and simple operation [36,37]. In ESPs, particles are charged in an electric field and then deposited on collecting electrodes [38,39].

Despite this potential, the use of ESPs in domestic boilers remains technically challenging. Small-scale systems have unstable gas flow, variable particle concentration and limited space for device installation, which shortens gas residence time and may reduce collection efficiency [4,40]. Efficiency may also decrease in the submicron “penetration window”, where particle collection is particularly difficult [41]. Additional processes, including particle coagulation, electrohydrodynamic flow and turbulence, may influence particle behavior inside the ESP [42]. Therefore, different compact, low-cost and micro-scale ESP designs have been developed and tested for small domestic boilers [43,44,45]. Recent studies indicate that such devices can substantially reduce PM emissions under laboratory and real-life conditions [24,46]. However, further improvements are still needed, particularly for small-scale biomass boilers burning fuels with variable properties [47,48,49].

Although PM emissions from conventional wood pellets and the performance of small-scale ESPs have been investigated previously, evidence remains limited regarding how fuel species affects both pollutant formation and ESP performance during the combustion of SRC pellets under identical domestic-boiler operating conditions. In particular, direct comparative studies that simultaneously evaluate PM emissions, CO emissions, and the effectiveness of electrostatic precipitation for different SRC feedstocks are scarce. This distinction is important because a pellet fuel associated with relatively low PM emissions may still produce high CO emissions. Therefore, the objective of this study was to determine particulate matter and carbon monoxide emissions from the combustion of pellets produced from three SRC species—willow, poplar, and black locust—in a domestic boiler before and after the application of an ESP. The results were compared with emissions obtained under analogous conditions during the combustion of conventional pine and birch wood pellets. The scientific novelty of this study lies in its controlled, species-specific comparison of alternative SRC pellets and conventional wood pellets in the same combustion system, both with and without an ESP, while jointly assessing PM and CO emissions. In addition, expressing emissions per unit of flue gas volume, fuel energy, and fuel mass enables a more comprehensive comparison of the PM- and CO-based emission performance of the tested fuels. This approach distinguishes fuel-related effects from ESP-related effects and provides new evidence for evaluating SRC pellets on the basis of their emission profiles.

## 2. Results and Discussion

### 2.1. Concentration-Based Emissions and Total Combustion Efficiency

PM and CO emissions were significantly affected by pellet species, although for CO this applied only to concentration-based emissions, whereas mass- and energy-based CO emissions were not statistically significant (Table 1). The application of the electrostatic precipitator had a significant effect only on PM emissions. The lack of a significant effect of the electrostatic precipitator on CO emissions from pellet combustion was also reported by Kantová et al. [50]. This was not surprising, because in the case of wood pellets, a significant reduction in CO emissions was observed after the use of a catalyst, rather than an electrostatic precipitator [21]. Other studies have also shown that the operation of an electrostatic precipitator may generate CO at levels of up to 30 ppm, and this phenomenon was correlated with the intensity of the corona discharge [48]. Only the interaction between species and the electrostatic precipitator was significant for both of these parameters.

The significantly highest PM emission was obtained from the combustion of willow pellets (64.12 mg m^−3^ at 10% O_2_; homogeneous group “a”) (Table 2). The lowest mean PM emission was recorded for pine pellets, corresponding to approximately 43% of the emission level observed for willow pellets; however, the difference between pine and birch should be interpreted in light of their shared homogeneous group. Among the SRC biomass pellets, poplar performed the most favorably; its emission factor was only slightly higher, by 1.61 mg m^−3^, compared with birch pellets, one of the most popular pellets produced from deciduous tree species. The application of the electrostatic precipitator significantly reduced PM emissions by approximately 53% (53.81 vs. 25.54 mg m^−3^). When PM emissions were broken down into individual interaction levels, the difference between the extreme combinations was almost 420% (W × NE = 90.96 mg m^−3^; P × WE = 17.51 mg m^−3^). Among the SRC pellets, poplar with the electrostatic precipitator performed the best, although its emission was still almost 34% higher than that of the most favorable variant.

The lowest PM emissions achieved during pellet combustion with the use of an electrostatic precipitator for pine, birch and poplar pellets (17.51, 19.94 and 23.43 mg m^−3^, respectively) were comparable with PM emissions from wood pellet combustion in a 20 kW pellet boiler operating at full load (21.1 mg m^−3^) [10]. Interestingly, in that study, such low emission levels were achieved without an electrostatic precipitator, which may have resulted, among other factors, from the more favorable composition and properties of the pellets, which met the ENplus standard requirements, including chemical composition, moisture content, length, mechanical durability and fines content. In other studies, spruce pellets combusted in an 18 kW boiler produced emissions of 18.83–26.82 mg m^−3^ without an electrostatic precipitator and 2.36–4.53 mg m^−3^ with an electrostatic precipitator [50]. Higher emissions for premium wood pellets were reported during combustion in a 150 kW boiler, reaching 37.2 mg m^−3^; however, this value was to some extent underestimated, as it referred to particles with a diameter of less than or equal to 2.5 µm [51], whereas total particulate matter emissions were determined in our study.

The differences in PM emissions among the tested pellets can be explained by the coexistence of several particle-formation pathways. Coarse particles are formed mainly through the mechanical entrainment of ash fragments, unburned char, and pellet fines from the fuel bed. In contrast, fine inorganic particles originate from ash-forming elements that are released into the gas phase at elevated temperatures. Volatile potassium- and sodium-containing compounds may subsequently undergo nucleation and condensation during flue-gas cooling, followed by coagulation and agglomeration. Chlorine can promote the volatilization of alkali metals, whereas sulfur affects their conversion into sulfate forms. Silicon- and aluminum-containing minerals may retain potassium in less volatile and more thermally stable compounds, thereby reducing its transfer to the fine particulate fraction [52,53]. Particulate matter may also contain soot, unburned carbon, and condensable organic compounds formed during incomplete oxidation of volatile fuel-decomposition products. The formation of this carbonaceous fraction is promoted by locally fuel-rich conditions, insufficient air–fuel mixing and insufficient residence time in the high-temperature oxidation zone [53,54]. Consequently, PM emissions cannot be predicted from total ash content alone because they depend on ash composition and speciation, mineral volatility, pellet fines, fuel-bed conditions, combustion temperature, and the efficiency of volatile-gas burnout.

The particularly high PM emissions from willow may therefore have resulted from a greater release or entrainment of ash-forming material under the applied combustion conditions. In contrast, the relatively low PM emissions from poplar suggest a lower transfer of mineral matter to the particulate fraction or more favorable particle retention in the fuel bed. However, because the concentrations of individual ash-forming elements and the chemical and size composition of the emitted particles were not determined, these mechanisms should be regarded as scientifically plausible explanations rather than as direct evidence of the chemical origin of PM in the present research.

In terms of CO emissions, pine and birch pellets also performed the most favorably, forming group “c” (220.85 and 216.79 mg m^−3^, respectively) (Table 2). In contrast, the significantly highest CO emission was recorded for poplar (2652.47 mg m^−3^, group “a”), which was 12 times higher than the emissions from the pellets included in group “c”. Among SRC pellets, willow and black locust (group “b”) showed lower CO emissions than poplar; however, their values remained approximately six times as high as those recorded for pine and birch (group “c”). As mentioned earlier, the application of the electrostatic precipitator did not significantly affect CO emissions. Analysis of the individual S × E combinations, for which the interaction was significant, showed that species determined CO emissions more strongly than the application of the electrostatic precipitator. The highest emission, obtained for poplar without the electrostatic precipitator, was more than 2569 mg m^−3^ higher than the lowest emission, recorded for birch with the electrostatic precipitator.

CO emissions from wood pellet combustion reported in the literature vary depending on boiler design, load, combustion control and operating phase. Jach-Nocoń et al. [10] reported CO concentrations of several hundred mg m^−3^ for wood pellet combustion in a 20 kW boiler, whereas Juszczak [55] indicated that modern pellet boilers equipped with oxygen-based control systems can achieve much lower CO concentrations. Other studies have shown that CO emissions are strongly dependent on air-flow control and the stability of pellet feeding [56,57,58]. One ENplus A1-class wood pellet showed a CO emission level similar to that recorded for pine and birch pellets (Table 2), i.e., 176 mg m^−3^ [59].

Carbon monoxide is formed as an intermediate product during the decomposition and oxidation of biomass, including volatile-matter combustion, char gasification, and the partial oxidation of hydrocarbons and oxygenated organic compounds. Its final concentration in the flue gas reflects the balance between CO formation and its subsequent oxidation to CO_2_. Efficient CO burnout requires adequate oxygen availability, effective mixing of combustible gases with air, and sufficient residence time in the high-temperature oxidation zone. An insufficient air supply creates locally oxygen-deficient regions, whereas excessive air may cool and dilute the combustion gases and thereby slow the oxidation of CO [53]. The physical properties of pellets can indirectly affect these processes. Differences in pellet dimensions, bulk density, mechanical durability, and fines content may modify the amount and temporal uniformity of fuel delivered by the feeding system. They can also influence fuel-bed porosity, oxygen penetration, heating rate, and the rate of volatile-matter release. A rapid release of volatile compounds that is not accompanied by an adequate secondary-air supply may create locally fuel-rich zones in which CO formation exceeds its subsequent oxidation to CO_2_. The high CO emissions from poplar therefore suggest that its devolatilization and combustion behavior were not optimally matched to the fixed fuel-feeding and air-supply settings used in the experiment. Conversely, the substantially lower CO emissions from pine and birch indicate more stable combustion and more complete oxidation of volatile compounds under the same operating conditions [53,54].

Fuel mineral composition may additionally affect CO oxidation. Gas-phase potassium compounds, particularly KCl and KOH, can interfere with the radical pool involved in CO oxidation, including reactions involving OH radicals. Experimental and kinetic studies have shown that potassium compounds may inhibit CO conversion, whereas potassium sulfation can partly modify or reduce this effect [60,61]. Because potassium speciation was not determined in the tested pellets, this pathway cannot be directly confirmed for the present experiment but represents a possible chemical contribution to the species-dependent CO emissions.

Regarding the total combustion efficiency of the analyzed pellets, it ranged from 90.20 to 92.57%, depending on the species (Table 2). The significantly highest value was achieved by black locust, which was the only species assigned to group “a”. The remaining combusted pellets were classified into group “b”. Both factors, species and electrostatic precipitator, significantly differentiated combustion efficiency, but independently of each other, as confirmed by the lack of a significant interaction. Among all variants, the highest efficiency was achieved for black locust with the electrostatic precipitator (93.20%). The difference between this variant and the one with the lowest efficiency, birch without the electrostatic precipitator, was 3.33 percentage points.

The obtained range of total combustion efficiency was similar to the values reported for modern small-scale pellet boilers. Jach-Nocoń et al. [10] and Verma et al. [62] indicated that modern pellet boilers can achieve efficiencies of approximately 90% or higher; however, these values depend on the species, fuel quality, burner design and combustion conditions. In general, efficiency and heat losses in a pellet boiler depend, among other factors, on excess air and the oxygen content in the flue gas [63]. The total combustion efficiency of small-scale pellet boilers can be improved, for example, by using pellets with low moisture and ash contents and by keeping heat-exchange surfaces clean, as these measures reduce chimney losses and losses due to incomplete combustion [1,10].

### 2.2. Energy-Based Emissions

Mean PM emissions with respect to species were divided into only two homogeneous groups. It should be emphasized that the relative grouping changes depending on the normalization basis, but this does not eliminate the unfavorable PM behavior of willow. Group “a”, with the highest emission, included only willow (43.26 mg MJ^−1^), whereas the remaining species had emissions approximately 39–51% lower and were classified into group “b” (Table 3). This indicates that, in terms of emissions per unit of energy, SRC pellets performed more favorably than in the case of emissions per unit of flue-gas volume, where four homogeneous groups were distinguished and no SRC pellet was assigned to the group characterized by the lowest emission (Table 2). Among the SRC pellets, poplar achieved the lowest value, similar to pine, one of the most popular species used for pellet production in Europe (22.54 mg MJ^−1^). The significant reduction in emissions after the application of the electrostatic precipitator was approximately 54% (17.52 vs. 38.11 mg MJ^−1^), which was therefore similar to the reduction observed for emissions expressed per unit of flue-gas volume. The reduction resulting from the use of the electrostatic precipitator was greatest for the most emissive willow pellet, reaching approximately 61%. The application of the electrostatic precipitator also reduced emission variability, expressed as the standard deviation, as clearly illustrated by the comparison of species with and without the electrostatic precipitator.

The change in the emission profiles after conversion from mg m^−3^ to mg MJ^−1^ results from the different reference bases of these metrics. Concentration-based values describe the amount of pollutant in a unit volume of flue gas, whereas energy-specific factors relate the emitted pollutant mass to the energy supplied by the fuel. The conversion therefore accounts for both the dry flue-gas volume and the fuel LHV. Fuels with higher LHV generally require less fuel to supply the same amount of energy, which may reduce their emissions expressed per MJ. Consequently, the energy-based ranking reflects the combined effects of pollutant concentration and LHV (indirectly also the fuel composition, which shapes, among others, LHV) [14]. Conversion to mg MJ^−1^ reduced the apparent differences among pellet species. Concentration-based emissions describe dry flue-gas quality, whereas energy-specific factors provide a more appropriate comparison of the pollutant intensity of fuels with different LHV and combustion-air requirements [52,53].

The lowest mean PM emission for pine pellets with the use of an electrostatic precipitator (13.75 mg MJ^−1^) was higher than that obtained for pellets also produced from a coniferous species, namely spruce, combusted during nominal operation of a 25 kW boiler (8.6 mg MJ^−1^; however, at lower load it increased to 21.4 mg MJ^−1^) [64]. In turn, in the study by Szyszlak-Bargłowicz et al. [65], PM emissions from wood pellets combusted in a 10 kW boiler amounted to 44.5 mg MJ^−1^. This emission level was higher than that recorded for all combinations in our experiment, except for willow without the electrostatic precipitator. An even higher PM emission level was obtained for agro-pellets made from rapeseed straw (1589 mg MJ^−1^) [65]. Apart from the use of an electrostatic precipitator, emissions from wood pellet combustion can also be reduced by applying Al–Si additives, especially kaolin, which in the study by Siegmund et al. [53] reduced PM emissions by approximately 49–50%. The mechanism involves binding K into stable K–Al–Si compounds in the ash, thereby limiting the formation of inorganic PM aerosols.

Poplar pellets, which performed the most favorably among the SRC pellets in terms of PM emissions, had the highest CO emission among all the tested pellets (1760.60 mg MJ^−1^, Table 3). Thus, when both dependent variables were considered, black locust was the most favorable SRC species under the tested conditions, as it was assigned to group “b” for the lowest PM emissions and, at the same time, to group “b” for intermediate CO emissions. The lowest emission levels were recorded for group “c”, consisting of pine and birch pellets (172.42 and 171.87 mg MJ^−1^, respectively), whereas in the above-mentioned intermediate group “b” (black locust and willow), emissions were higher by approximately 763–764 mg MJ^−1^. In some interaction variants, both decreases and increases in CO emissions were observed for individual pellet species when expressed per unit of energy; however, these differences were never significant. This agrees with the non-significant S x E interaction for energy-based CO emissions (Table 1). Species was the factor that significantly determined this emission indicator. Poplar pellets were also characterized by the greatest variability in CO emissions.

Mean CO emissions obtained for SRC pellets were similar to the emissions of 906–1293 mg MJ^−1^ reported for ENplus A1- and ENplus A2-class beech pellets combusted in an automatic pellet appliance with a nominal capacity of 8 kW [66]. Only poplar pellets deviated markedly from this range. In another study, the mean CO emission from wood pellets combusted in a 10 kW boiler was 760.7 mg MJ^−1^ [67], which, compared with the present study, was lower than that recorded for SRC pellets but higher than that obtained for the typical species used for pellet production, i.e., pine and birch. Moreover, Delcourt et al. [67] investigated the combustion of wood pellets and mixtures with barley straw pellets in a domestic biomass boiler. For the mixture containing 10% straw pellets, CO emissions were lower than for pure wood pellets, which resulted from the lower density of the straw pellets and a more favorable air-to-fuel ratio under constant volumetric fuel feeding. At a 50% share, CO emissions increased due to ash sintering and impaired air supply to the combustion zone. Although an electrostatic precipitator does not reduce CO emissions, catalytic systems installed in the flue-gas flow path may be considered a secondary method for reducing them. Gaze et al. [68] investigated TiO_2_, CuO, MnO_2_ and Pt catalysts in a 15 kW boiler fired, among others, with wood pellets. The study showed that the catalysts enabled CO oxidation in the flue gas; for the CuO system, CO reduction during wood pellet combustion amounted to 21%.

### 2.3. Emission Factors Expressed per Unit of Pellet Mass

Mass-based emission factors indicate the amount of pollutant released during the combustion of a defined mass of fuel and therefore have direct practical relevance for emission inventories and fuel-use planning. For example, multiplying an emission factor expressed in g Mg^−1^ by the annual mass of pellets consumed provides an estimate of the annual PM or CO load from a boiler. Values expressed per unit of dry matter additionally remove the effect of fuel moisture and enable comparisons among fuels with different water contents. In the present study, moisture varied only slightly, from 5.93 to 6.48% (Section 3.2.), which explains why conversion to a dry-mass basis increased the emission factors by a similar and relatively small proportion for all pellet types. Mass-based factors complement energy-specific indicators: they describe emissions associated with the quantity of fuel purchased and combusted, whereas energy-based factors describe emissions relative to the useful energy potential of the fuel.

The differences among pellet types resulted primarily from their pollutant concentrations and from the flue-gas volume generated per unit mass of fuel, which depends on elemental composition and stoichiometric air demand. Willow had the highest PM factor (Table 4) because its combustion produced the highest PM concentration (Table 3), indicating greater entrainment or formation of ash-derived particles under the applied conditions. The SRC pellets also contained substantially more ash (1.58–1.85% d.m.; Section 3.2.) than pine and birch (0.19 and 0.40% d.m.), increasing the amount of mineral matter potentially available for particle formation, although ash composition and volatility are also important. Poplar produced relatively low PM (Table 4) but the highest CO (Table 5) factor because its exceptionally high CO concentration (Table 3) indicated incomplete oxidation of volatile compounds. By contrast, pine and birch combined low ash contents, higher volatile-matter contents, and higher LHV with much lower CO concentrations (Section 3.2. and Table 3), suggesting more stable combustion and more complete burnout [52].

The mean PM emission expressed per unit of pellet mass was significantly highest for willow pellets (747.90 g Mg^−1^, group “a”), whereas the remaining pellets were assigned to group “b”, characterized by significantly lower emissions (Table 4). Emissions in this group were lower by 294–364 g Mg^−1^. The application of the electrostatic precipitator significantly reduced emissions by approximately 358 g Mg^−1^ (54%). The most favorable variant, i.e., pine with the electrostatic precipitator (247.64 g Mg^−1^), was characterized by emissions more than four times lower than those of the least favorable variant, willow without the electrostatic precipitator (1079.91 g Mg^−1^). When expressed on a dry-mass basis, the assignment of homogeneous groups to individual factor levels and interactions was identical. However, in each case, PM emissions increased because conversion to dry mass eliminates the share of moisture contained in the pellets, thereby reducing the reference mass while the amount of emitted substances remains unchanged.

The mean PM emissions obtained without the use of an electrostatic precipitator for pellets produced from dedicated forest dendromass species (555.61 and 660.33 g Mg^−1^ d.m.) were lower than those reported for pellets from another conventional forest biomass pellets, namely spruce (797 g Mg^−1^ d.m.; boiler with a nominal capacity of 10 kW) [65]. Two of the tested SRC pellets also achieved lower emissions, i.e., poplar and black locust, at 546.36 and 622.76 g Mg^−1^ d.m., respectively, whereas only willow differed unfavorably from spruce, with emissions higher by 351 g Mg^−1^. Rapeseed straw agro-pellets were characterized by a much higher emission level, i.e., 23,282 g Mg^−1^ d.m. [65], which was 1928–4090% higher than that recorded for the entire group of tested wood pellets. In another study [69], combustion of wood pellets produced from logging residues in a stove with a power range of 3–10 kW resulted in emissions of 462–1411 g Mg^−1^, without an electrostatic precipitator and without conversion to fuel dry mass. At high load, the emission was the lowest and, at the same time, lower than that recorded for all species combusted without the electrostatic precipitator in the present study. Apart from the above, it should be emphasized that even during the combustion of pellets produced from the same species and belonging to the same quality class, PM emissions may vary depending on the fuel producer and boiler design [1].

As regards CO, the mean emission per unit of pellet mass for poplar was significantly the highest, both without the electrostatic precipitator (31,444 g Mg^−1^) and with the electrostatic precipitator (29,669 g Mg^−1^) (Table 5). These two interaction levels were the only ones assigned to homogeneous group “a”. The intermediate group “b” consisted of the four remaining SRC pellet variants, including willow and black locust, whose combustion emitted approximately 259–815% more CO than the four lowest-emission variants, represented by pine and birch. When only CO emissions are considered, willow and black locust showed lower values among the SRC pellets than poplar. However, the lowest CO emissions among all tested treatments were recorded for conventional forest biomass pellets, particularly pine and birch. At the species level, pine and birch were characterized by the lowest mean CO emissions among all five pellet types, at 3104.16 and 2985.70 g Mg^−1^, respectively. When expressed on a fuel dry-mass basis, these mean CO emission factors increased in both cases by approximately 6.9%, which resulted from the similar moisture content of the two pellets.

Vicente et al. [21] reported CO emission factors of 9280 g Mg^−1^ d.m. for locally purchased commercial wood pellets combusted in an automatically fed pellet stove with a nominal heat capacity of 9.6 kW, without additional emission reduction devices, operated at nominal load. This emission level was therefore higher than that recorded for pine and birch in the present study, but lower than that obtained for the tested SRC pellets. After conversion to a dry-mass basis, the CO emission factors reported by Matijasevic et al. [66] for beech pellets, combusted in an automatic residential pellet stove with a nominal heat output of 8 kW and without additional emission reduction devices, corresponded to approximately 23,960 g Mg^−1^ d.m. for A1-class pellets. This indicates that beech pellets performed more favorably in this respect only when compared with poplar pellets. CO emissions are strongly affected by the content of potassium and other mineral components, because KOH and KCl compounds may inhibit CO oxidation in the gas phase by influencing the radical pool. Moreover, CO emissions depend on combustion temperature, gas residence time in the high-temperature zone and the intensity of fuel–air mixing [52].

### 2.4. General Characteristics of the Pellets and Their Profiles

Table 6 presents descriptive statistics for the entire dataset, including all analyzed combinations of combusted pellets. The data indicate that the lowest variability, expressed as the coefficient of variation, was found only for η, i.e., 1.24%. In contrast, the remaining qualitative and quantitative traits were characterized by much higher variability, ranging from 50.63 to 78.61%. Variability in PM was consistently lower than variability in CO. Expressing emission factors originally given per unit of flue-gas volume in relation to pellet energy and mass always reduced variability, with the greatest reduction observed after conversion to pellet mass.

Species accounted for the variability in CO and η (Figure 1). In contrast, PM was modified by both species and the electrostatic precipitator, with the contribution of the electrostatic precipitator being greater in this case. The contribution of the electrostatic precipitator to PM reduction increased when emissions expressed per unit of flue-gas volume were converted to emissions per unit of pellet energy and mass.

The PCA was used as an exploratory tool to visualize the multivariate relationships among the parameters examined during pellet combustion (Figure 2). No clear strong relationships among the analyzed traits were apparent from this exploratory projection. In practical terms, the most desirable variant should combine high or acceptable combustion efficiency with the lowest possible emissions of both PM and CO. The PCA scores, interpreted together with the direct emission and efficiency results, suggested that W × NE represented the least favorable variant, as it was positioned closest to the PM-related direction and was also associated with relatively low total combustion efficiency. Variants located closer to the CO direction were also less favorable from an emission perspective, particularly those involving P × NE and BL × NE. Among the SRC pellets, BL × WE showed the most favorable overall position, which was consistent with its high combustion efficiency and relatively low PM and CO emissions. The application of the electrostatic precipitator generally shifted the variants toward regions of the PCA space associated with lower PM emissions and higher efficiency. However, this pattern should be interpreted as a descriptive visualization of the observed dataset rather than as independent confirmatory evidence. Considering only the emission indicators, the most favorable combinations were pine and birch with the electrostatic precipitator (PI × WE and B × WE), which combined the lowest PM and CO emissions, although their combustion efficiencies were among the lowest of the variants tested with the electrostatic precipitator (90.36% for PI × WE and 90.53% for B × WE, Table 2).

Hierarchical clustering was also used as an exploratory method to describe similarities among the emission and combustion profiles of the analyzed variants (Figure 3). The dendrogram suggested that the overall profile of pellet combustion differed depending on both biomass species and the application of the electrostatic precipitator, with willow pellets combusted without the electrostatic precipitator (W × NE) forming the most distinct variant in the analyzed dataset. This variant joined the remaining samples only at the greatest linkage distance, which reflected its specific combination of high PM emissions and relatively low efficiency. The closest similarity was observed between pine and birch pellets, especially in the variants with the electrostatic precipitator (PI × WE and B × WE), suggesting comparable emission and combustion patterns under the tested conditions. Poplar and black locust occupied an intermediate position in the clustering structure. The relatively close grouping of P × NE with P × WE and BL × NE with BL × WE suggested that, for these species, the electrostatic precipitator reduced the particulate fraction but did not fully alter the overall emission profile. These clustering results should therefore be treated as an exploratory summary of similarities within the present dataset. They support the interpretation that a reduction in PM emissions alone should not be regarded as unequivocal improvement in the entire combustion process or emission profile. In general, the final assessment of pellet emissivity should consider at least both PM and CO emissions, because only simultaneously low values of these two parameters may indicate a potentially environmentally acceptable combustion profile.

It should also be noted that the multivariate patterns and statistical groupings were obtained from a balanced but relatively small experimental dataset, with three replications per treatment combination. Therefore, these patterns should be interpreted primarily as an indication of emission-profile differences under the controlled conditions of the present boiler test rather than as a definitive generalization for all combustion systems and operating regimes.

### 2.5. Study Limitations

This study has several limitations that should be considered. The combustion tests were performed using one modern small-scale pellet boiler and one integrated electrostatic precipitator design; therefore, the results should not be directly generalized to all boiler types, burner designs or ESP configurations. The measurements were conducted under controlled conditions and relatively short measurement windows, which may not fully represent long-term real-life operation, including start-up, shutdown, fouling, ash accumulation and changes in ESP performance over time. The emission assessment was limited to particulate matter and carbon monoxide, whereas other pollutants, PM chemical composition and organic or inorganic trace compounds were not determined. Consequently, the findings should be interpreted as a controlled comparison of PM and CO emission behavior of the tested fuels in the applied boiler–ESP system.

## 3. Materials and Methods

### 3.1. Experimental Setup

Emission tests were conducted using an experimental setup equipped with an Eko Balance pellet boiler with a nominal output of 24 kW and an integrated electrostatic precipitator (PPUH ZAMECH, Czeladź, Poland); a self-cleaning pellet burner; a fuel tank with a capacity of 300 dm^3^; a pellet feeding system; a controller regulating boiler operation, manufactured by TechSterowniki (Wieprz, Poland); a Testo 300 flue-gas analyzer, used, among others, to measure O_2_ and CO, chimney draft and flue-gas temperature; a Testo 380 + 330 particulate matter measuring system (Testo SE & Co. KGaA, Titisee-Neustadt, Germany), used, among others, for simultaneous real-time measurement of particulate matter, CO and O_2_ concentrations; a flue duct; and a computer for data archiving. The measuring stand was also equipped with instrumentation enabling control of the combustion process, including a Wilo pump for automatic and manual regulation of water flow in the installation; an RSHT chimney fan with an EBC24 module (Exodraft, Langeskov, Denmark), allowing a constant set pressure of 15 Pa to be maintained in the flue duct/chimney; a UPS-2000SE uninterruptible power supply (AZO Digital Sp. z o.o., Chwaszczyno, Poland); and Volcano heaters (VTS Polska Sp. z o.o., Gdańsk, Poland) necessary for heat removal from the water circulation system.

The boiler met the ecodesign requirements specified in Commission Regulation (EU) 2015/1189 of 28 April 2015 implementing ecodesign requirements for solid fuel boilers, as well as the requirements of class 5 according to PN-EN 303-5:2012 [70], which were in force at the time of its certification/testing.

A commercial electrostatic precipitator (ESP) designed to reduce particulate matter emissions from biomass-fired residential boilers was used in the experiments. The ESP was installed in the flue-gas duct of an automatic Eko Balance pellet boiler. The device consisted of a discharge electrode, a mounting rod with a ceramic insulator, an acid-resistant stainless-steel housing, and a power supply equipped with a high-voltage generator. The discharge electrode, which generated corona discharges, was made of steel wire with sharpened tips and was positioned centrally within the flue-gas duct. The grounded walls of the flue-gas duct served as the collecting electrode, on which the electrically charged particulate matter was deposited.

The ESP system was operated by a controller supplied with 230 V AC at 50 Hz. The controller continuously monitored the flue-gas temperature and automatically activated the high-voltage system when the flue-gas temperature reached 40 °C. The switching hysteresis was set at 2 °C, while the high-voltage generator operated at a frequency of 18 kHz.

Before the measurements, the Eko Balance boiler was thoroughly cleaned and then equipped with the research instrumentation. The combustion chamber was also carefully cleaned each time the pellet species was changed. The following components were also inspected: the pellet burner, responsible for proper combustion; the self-cleaning burner grate, which ensures proper combustion; the photocell, enabling the burner to switch from ignition mode to operation mode; the igniter, i.e., the ceramic heating element responsible for pellet ignition; the controllers; the exhaust fan, which maintains the constant chimney draft required for proper emission measurements; and the Testo 300 measuring equipment and the Testo 380 + 330 particulate matter measuring system, as the amount of gaseous substances and particulates released from the boiler into the flue duct was measured in each case.

All measurements were performed with the boiler operating at its nominal load. Due to differences in the properties of the tested pellets, the combustion parameters, including the fuel feed rate and combustion air supply, were individually adjusted for each fuel. For pine pellets, the operating conditions were similar to those specified in the boiler certification test report issued by an accredited laboratory. Under nominal-load conditions, this corresponded to a thermal output of 24.3 kW and a fuel feed rate of 5.28 kg h^−1^.

In the present study, the boiler settings were optimized separately for each pellet type. These parameters were adjusted manually to achieve the highest possible combustion stability and proper burner operation. Once the optimal operating parameters had been established for a given pellet type, no further adjustments were made during the emission measurements. During the measurements, the oxygen concentration in the flue gas ranged from 10.17 to 13.64%, the excess air ratio (λ) from 1.93 to 2.85, the combustion air volume from 8.80 to 13.00 m^3^ kg^−1^ of fuel, and the flue-gas temperature from 87.5 to 99.5 °C.

### 3.2. Combustion Process and Emission Measurements

All pellet species were stored in the same manner, i.e., in bags placed in a designated area of a brick warehouse, without exposure to excess moisture. The composition and properties of the pellets used in this study were known, as they had been determined in a previous study (in the present study, pellets from exactly the same batches were used) (Table 7) [14].

At the start of the combustion process, each pellet species was weighed using an electronic scale, and the prepared fuel, in the amount of 150 kg, was loaded into the boiler hopper. The boiler, fan and exhaust fan were then started using the controller. A natural stage of boiler operation was the transition from the ignition procedure to automatic operation. In each case, the purpose of boiler operation was to achieve a stable temperature of the water supplying the heating system of 65–69 °C, while the water returning from the installation reached a target temperature of 56–60 °C. Once stable boiler operation had been achieved, the measuring equipment was started. The time required for these devices to become ready for operation was approximately 20 min and included probe heating, calibration and zeroing. After this period, the probes were placed in the chimney located between the boiler flue outlet and the flue duct, precisely 1 m from the flue-gas outlet. The sampling point was located within a 1.5 m straight section of the flue-gas duct to provide sufficiently developed and stable flue-gas flow and adequate mixing of the gaseous components and particulate matter prior to measurement. The sampling location was selected away from bends, duct connections, and other elements that could generate local flow disturbances or concentration gradients. It was also positioned outside the zones directly affected by the boiler outlet and the particulate removal system. These conditions were intended to minimize flow non-uniformity and to ensure that the sampled flue gas was representative of the gas stream under the tested operating conditions.

The prepared measuring system enabled real-time flue-gas measurements and the collection of all data using the EasyHeat software (Testo SE & Co. KGaA, Titisee-Neustadt, Germany). Particulate matter, CO, and O_2_ concentrations were continuously monitored for at least 30 min for each pellet type and each ESP operating condition. From the recorded time series, 10 min intervals corresponding to stable boiler operating conditions and stable pollutant concentration profiles were selected for statistical analysis. The temporal stability of PM concentrations within the selected 10 min intervals was assessed using the coefficient of variation (CV%), calculated from individual measurements recorded at 5 s intervals. The CV values ranged from 7.2% to 16.8%, with a mean value of 11.4%. In total, 30 measurements were performed, comprising combinations of five pellet species and two configurations, with three replications. After completing the full cycle for a single combination, the ash chamber, hopper and feeding system were always cleaned, and the test stand was fully prepared for the next experimental combination.

PM and CO emissions were expressed as mg m^−3^ in dry flue gas, under standard conditions, i.e., 101,325 Pa and 273.15 K, and converted to a reference O_2_ concentration of 10% according to the following equation:

(1) $$c_{ref}=c_{meas}\frac{\left(20.95-\;O_{2\;ref}\right)}{\left(20.95-O_{2\;meas}\right)}\;$$

where:

*c_ref_*—PM or CO concentration normalized to the reference oxygen concentration (mg·m^−3^),

*c_meas_*—measured PM or CO concentration (mg·m^−3^),

*O*_2 *ref*_ —reference oxygen concentration (10% for small-scale pellet boilers),

*O*_2 *meas*_—measured oxygen concentration in the flue gas (%).

Total combustion efficiency, *η* (%), was calculated, as well as gas and particulate emissions expressed per unit of pellet energy, *EF* (mg MJ^−1^), and emissions per unit of pellet mass, *EF′* (g Mg^−1^ at the respective pellet moisture content and g Mg^−1^ on a dry-mass basis, d.m.). Total combustion efficiency accounted for chimney loss (*q_A_*), i.e., energy lost with hot flue gas, and incomplete combustion loss (*q_U_*), resulting from the presence of CO, meaning that part of the carbon from the fuel was not completely oxidized to CO_2_. The calculations were performed using the following equations:

(2) $$\eta =100-q_{A}-q_{U}$$

(3) $$q_{A}=\left(T_{gas}-T_{amb}\right)\left(\frac{A}{CO_{2\%}+B}\right)$$

(4) $$q_{U}=\frac{\alpha \;CO_{\%}}{CO_{\%}+CO_{2\%}}$$

where:

*q_A_*—flue-gas heat loss (chimney loss) (%),

*q_U_*—heat loss due to incomplete combustion (%),

*T_gas_*—flue-gas temperature (K),

*T_amb_*—ambient temperature (K),

*CO*_2%_ and *CO*_%_—concentrations of CO_2_ and CO in the flue gas (%),

*A*, *B*—Siegert coefficients (*A* = 0.65, *B* = 0),

*α*—fuel-specific coefficient (*α* = 0.69).

In turn, the emission factors (*EF* and *EF′*) were calculated based on combustion stoichiometry methodology [1,71,72,73] using the following equations:

(5) $$EF\prime =\frac{c_{ref}\;V_{DE}\;20.95\;}{\left(20.95-O_{2\;ref}\right)}$$

(6) $$EF=\frac{EF\prime }{LHV}$$

(7) $$V_{DE}=V_{CO2}+V_{N2}+V_{O2}+V_{SO2}$$

(8) $$V_{DE}=1.89\;\frac{C}{100}+0.81\;\frac{N}{100}+0.79\;V_{air\;s}+0.21\;\left(\lambda -1\right)\;V_{air\;s}+0.71\;\frac{S}{100}$$

(9) $$V_{air\;s}=3.336\;\frac{2.667\;C+8\;H+S-O}{100}$$

(10) $$\lambda =\frac{20.95}{20.95-O_{2\;\mathrm{meas}}}$$

where:

*V_DE_*—volume of dry flue gas (m^3^ kg^−1^),

*V_CO_*_2_, *V_N_*_2_, *V_O_*_2_ and *V_SO_*_2_—volumes of the main gaseous components in the flue gas, i.e., CO_2_, N_2_, O_2_, and SO_2_, respectively (m^3^ kg^−1^),

*V_air s_*—stoichiometric volume of air required for combustion (m^3^ kg^−1^),

*λ*—excess air ratio,

*C*, *H*, *S*, *N*, and *O*—elemental contents in the fuel (%).

The elemental composition and thermophysical parameters of the tested pellets required for the calculations are presented in Table 7.

### 3.3. Statistical Analyses

For the tested traits, parameters of descriptive statistical analysis were calculated: mean, median, minimum value (Min), maximum value (Max), lower quartile (Q1), upper quartile (Q3), standard deviation (SD) and coefficient of variation (CV). The normality of the traits was verified using the Shapiro–Wilk test and the homogeneity of variance using Levene’s test, and a two-way analysis of variance (ANOVA) was performed (STATISTICA 13, TIBCO Software Inc., Palo Alto, CA, USA). Because three replications were available for each species × electrostatic precipitator combination, the results of formal assumption tests were interpreted with caution. In small treatment groups, tests such as Shapiro–Wilk have limited power to detect departures from normality. Therefore, statistical conclusions were evaluated together with descriptive statistics, effect magnitudes, and the consistency of trends across the different emission metrics. Arithmetic means and standard deviations were calculated for all parameters. Homogeneous groups were identified using Tukey’s HSD multiple-comparison test at a significance level of *p* < 0.05. To determine the proportion of trait variability explained by the main factors and their interactions, their percentage contributions to the total sum of squares were calculated. The percentage contribution of each factor was calculated from the sums of squares obtained using sigma-restricted parameterization and effective hypothesis decomposition. The design was balanced (with an equal number of replicates). Pearson correlation coefficients were determined between the analyzed parameters (*p* < 0.05). As part of the agglomerative hierarchical clustering analysis, the results were standardized by columns; Ward’s method was used as the agglomeration method, and Euclidean distance was used as the distance measure. A dendrogram was constructed to illustrate the similarities among the analyzed pellets. Principal component analysis was performed on standardized data, excluding variables calculated indirectly or representing subsets of other variables. The results were presented as variable correlation circles and case projections onto the PC1–PC2 plane.

## 4. Conclusions

Based on PM and CO emissions, pellets produced from short-rotation coppice (SRC) biomass showed emission suitability for use as a biofuel in the tested modern small-scale boiler; however, their emission characteristics depend primarily on the biomass species and the use of a flue-gas cleaning system. The electrostatic precipitator reduced the mean PM concentration by 52.5%, from 53.81 to 25.54 mg m^−3^, but did not significantly affect CO emissions (1214.99 and 1160.98 mg m^−3^ without and with the electrostatic precipitator, respectively). Biomass species significantly differentiated particulate matter and carbon monoxide emissions, confirming that SRC biomass pellets should not be treated as a homogeneous fuel group. Among the tested SRC pellets, willow pellets were characterized by the highest PM emissions (64.12 mg m^−3^), especially without the electrostatic precipitator (90.96 mg m^−3^), whereas poplar pellets showed relatively low PM emissions (33.88 mg m^−3^) but, at the same time, the highest CO emissions (2652.47 mg m^−3^). Therefore, the assessment of combustion quality and emission suitability based on PM and CO should include a combined analysis of at least PM and CO emissions.

The application of the electrostatic precipitator significantly reduced PM emissions in all fuel variants, regardless of how the emissions were expressed: in relation to flue-gas volume, energy unit or fuel mass. At the same time, the electrostatic precipitator did not significantly affect CO emissions. The electrostatic precipitator is therefore an effective secondary measure for reducing particulate matter emissions, but it does not compensate for incomplete combustion or for fuel properties responsible for CO formation. Reducing CO emissions requires the application of other methods, including primary measures, such as appropriate selection of pellet species and pellet quality.

The lowest emission levels were obtained for conventional pine and birch pellets combusted with the use of the electrostatic precipitator, especially when PM and CO emissions were considered simultaneously. Pine pellets produced PM and CO emissions of 17.51 and 228.64 mg m^−3^, respectively, whereas the corresponding values for birch pellets were 19.94 and 142.97 mg m^−3^. Among the SRC pellets, black locust pellets combusted with the electrostatic precipitator showed the most favorable emission–energy compromise, combining the highest combustion efficiency (93.20%) with relatively low PM and CO emissions (29.56 and 1322.20 mg m^−3^, respectively). This variant can be considered the most promising among the tested SRC pellets in terms of emission suitability based on PM and CO and combustion efficiency under the conditions of the boiler used. The least favorable variant was the combustion of willow pellets without the electrostatic precipitator, mainly due to high PM emissions (90.96 mg m^−3^) and lower combustion efficiency (90.00%).

Based on PM and CO emissions, the obtained results indicate that the use of alternative woody biomass pellets in individual heating may be possible, but it requires species-specific emission assessment rather than their simple substitution for conventional wood pellets. The electrostatic precipitator can clearly improve the emission profile of SRC pellets in terms of PM; however, the final environmental effect depends on the simultaneous control of at least both PM and CO emissions. Further research should focus on optimizing boiler settings for individual SRC fuels and on evaluating the long-term effectiveness of the electrostatic precipitator under variable real-life operating conditions.

These findings should be validated in other boiler designs, ESP configurations and real-world operating cycles.

## Figures and Tables

**Figure 1:**
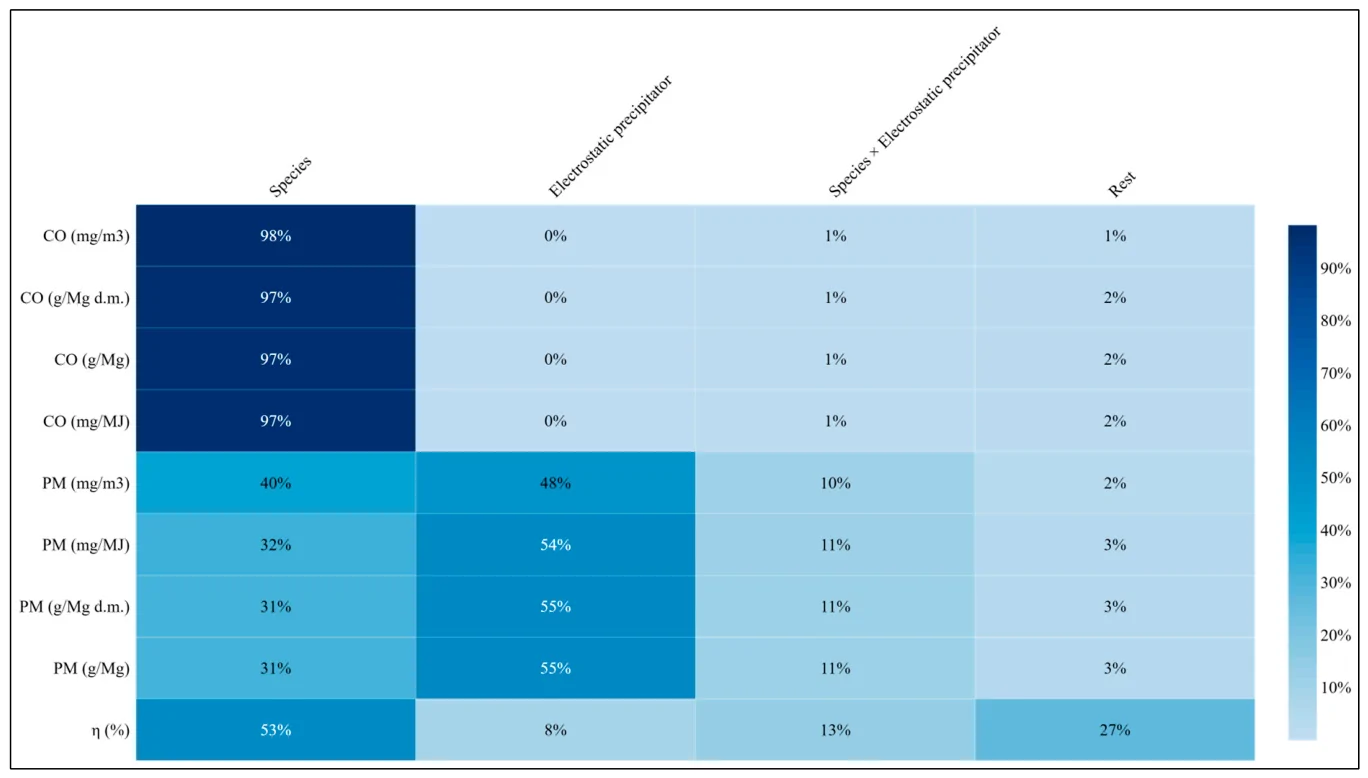
Heatmap showing the percentage contribution of main factors and their interactions to the total variance of analyzed pellets. Color intensity increasing with effect size. “Rest” denotes unexplained variance.

**Figure 2:**
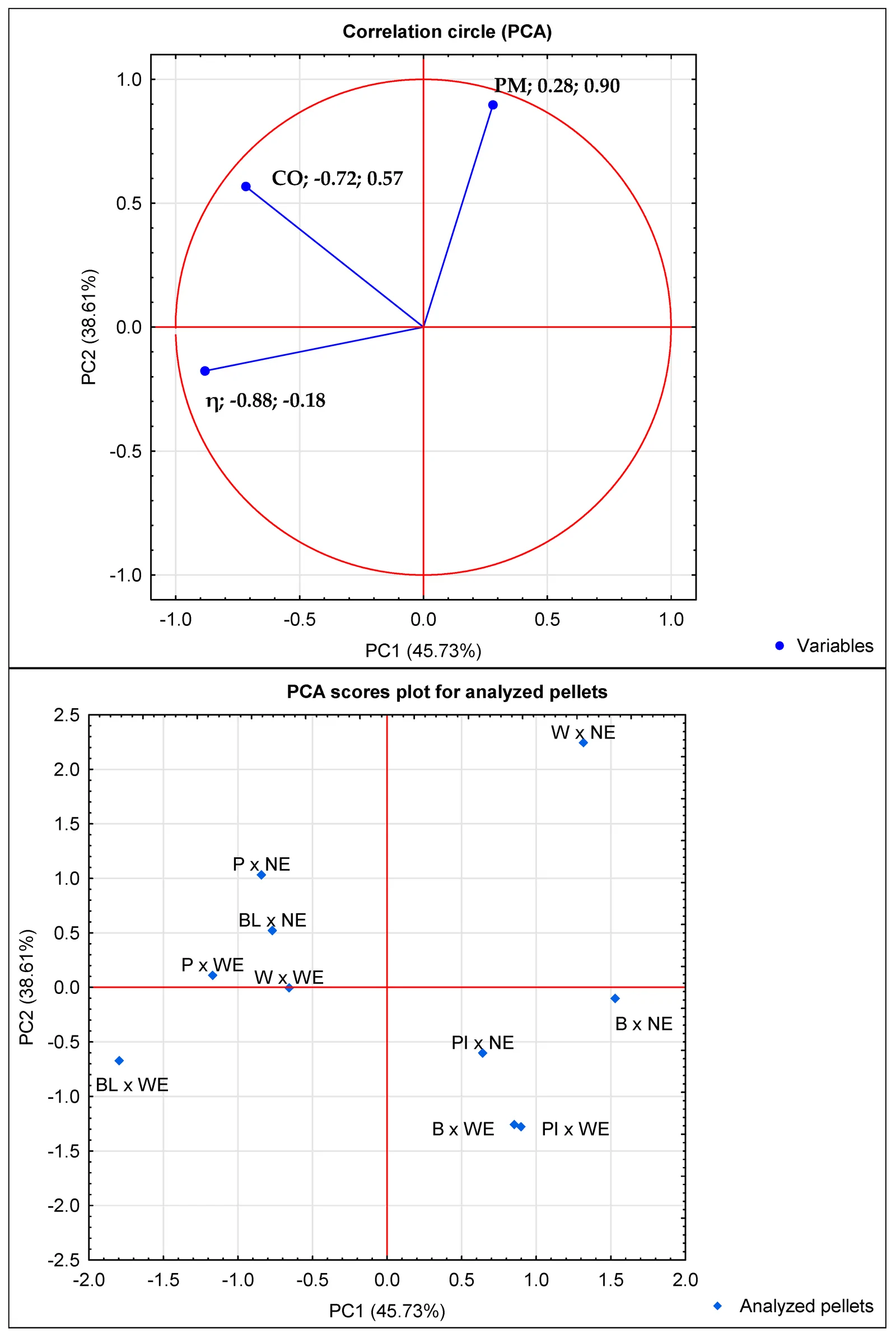
Principal component analysis (PCA) summarising relationships among analyzed pellet traits and differences among species × electrostatic precipitator. The upper panel shows the PCA correlation circle (loadings) for the analyzed variables. The lower panel presents the PCA scores for species × electrostatic precipitator. Notes: W = willow, P = poplar, BL = black locust, PI = pine, B = birch, NE = without ESP, and WE = with ESP.

**Figure 3:**
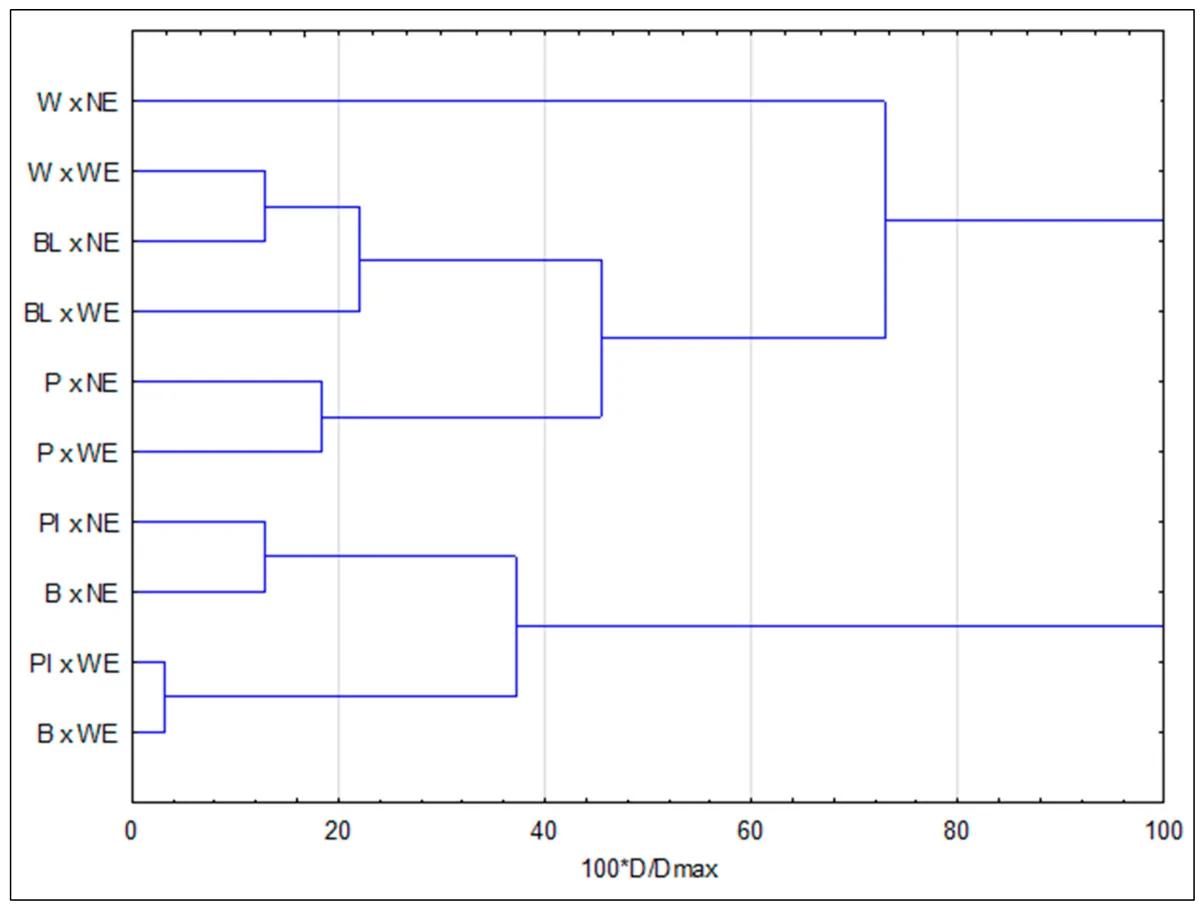
Dendrogram of hierarchical cluster analysis presenting mutual similarities of analyzed pellets. D—linkage distance; D_max_—maximum linage distance.

**Table 1:** Analysis of variance (*p* values) for the analyzed features (F-values are given in parentheses).

Attribute	Species (S)	Electrostatic Precipitator (E)	S × E
PM (mg m^−3^)	<0.001 * (96.887)	<0.001 * (465.263)	<0.001 * (23.827)
CO (mg m^−3^)	<0.001 * (482.254)	0.207 (1.701)	0.019 * (3.783)
η (%)	<0.001 * (9.9)	0.025 * (5.9)	0.079 (2.5)
PM (mg MJ^−1^)	<0.001 * (48.624)	<0.001 * (326.363)	<0.001 * (16.085)
CO (mg MJ^−1^)	<0.001 * (298.542)	0.082 (3.352)	0.107 (2.189)
PM (g Mg^−1^)	<0.001 * (45.495)	<0.001 * (322.013)	<0.001 * (15.397)
CO (g Mg^−1^)	<0.001 * (299.475)	0.082 (3.345)	0.106 (2.194)
PM (g Mg^−1^ d.m.)	<0.001 * (46.580)	<0.001 * (332.993)	<0.001 * (15.831)
CO (g Mg^−1^ d.m.)	<0.001 * (307.138)	0.078 (3.439)	0.099 (2.257)

* Significant values (*p* < 0.05), number of degrees of freedom = 29.

**Table 2:** PM and CO emissions and total combustion efficiency as affected by pellet species, electrostatic precipitator application, and their interaction.

Source of Variation	Item	PM (mg m^−3^) *	CO (mg m^−3^) *	η (%)
	Willow (W)	64.12 ± 29.51 a	1407.28 ± 148.75 b	90.81 ± 0.94 b
	Poplar (P)	33.88 ± 12.36 c	2652.47 ± 160.84 a	91.08 ± 0.41 b
Species (S)	Black locust (BL)	40.50 ± 12.30 b	1442.53 ± 191.26 b	92.57 ± 0.70 a
	Pine (PI)	27.62 ± 11.57 d	220.85 ± 57.10 c	90.69 ± 1.28 b
	Birch (B)	32.27 ± 13.67 cd	216.79 ± 83.29 c	90.20 ± 0.55 b
Electrostatic precipitator (E)	Without (NE)	53.81 ± 20.13 a	1214.99 ± 958.48	90.76 ± 1.14 b
	With (WE)	25.54 ± 7.58 b	1160.98 ± 941.41	91.38 ± 1.07 a
	W × NE	90.96 ± 2.35 a	1295.63 ± 121.53 b	90.00 ± 0.44 bc
	W × WE	37.28 ± 3.37 cd	1518.94 ± 56.12 b	91.63 ± 0.11 abc
	P × NE	44.33 ± 7.00 bc	2712.81 ± 229.17 a	90.97 ± 0.59 bc
	P × WE	23.43 ± 2.34 ef	2592.14 ± 35.12 a	91.20 ± 0.17 abc
S × E	BL × NE	51.43 ± 4.37 b	1562.85 ± 105.03 b	91.95 ± 0.28 ab
	BL × WE	29.56 ± 0.16 de	1322.20 ± 192.31 b	93.20 ± 0.08 a
	PI × NE	37.73 ± 5.15 cd	213.05 ± 88.90 c	91.02 ± 1.94 bc
	PI × WE	17.51 ± 1.22 f	228.64 ± 8.13 c	90.36 ± 0.16 bc
	B × NE	44.61 ± 2.79 bc	290.60 ± 23.50 c	89.87 ± 0.53 c
	B × WE	19.94 ± 1.59 ef	142.97 ± 21.08 c	90.53 ± 0.39 bc

±—Standard deviation; *—air pollutant concentration normalized to standard conditions (101,325 Pa, 273.15 K) and to a reference oxygen content of 10% O_2_ in flue gas; a, b, c, d, e, f—different lowercase letters indicate statistically significant differences between means (homogeneous groups; Tukey’s HSD test *p* < 0.05).

**Table 3:** PM and CO emissions expressed per unit of fuel energy as affected by pellet species, electrostatic precipitator application, and their interaction.

Source of Variation	Item	PM (mg MJ^−1^)	CO (mg MJ^−1^)
	Willow (W)	43.26 ± 21.15 a	934.93 ± 80.55 b
	Poplar (P)	22.54 ± 8.56 b	1760.60 ± 152.19 a
Species (S)	Black locust (BL)	26.37 ± 8.60 b	935.38 ± 139.78 b
	Pine (PI)	21.32 ± 8.98 b	172.42 ± 49.90 c
	Birch (B)	25.57 ± 11.17 b	171.87 ± 68.60 c
Electrostatic precipitator (E)	Without (NE)	38.11 ± 13.34 a	826.36 ± 630.57
	With (WE)	17.52 ± 3.99 b	763.72 ± 607.76
	W × NE	62.47 ± 2.50 a	890.33 ± 95.41 b
	W × WE	24.06 ± 2.37 cd	979.53 ± 33.92 b
	P × NE	29.61 ± 5.43 bc	1811.83 ± 220.90 a
	P × WE	15.47 ± 1.91 de	1709.38 ± 35.12 a
S × E	BL × NE	33.99 ± 3.21 b	1032.66 ± 78.41 b
	BL × WE	18.74 ± 0.19 de	838.09 ± 119.60 b
	PI × NE	28.89 ± 5.39 bc	165.22 ± 77.72 c
	PI × WE	13.75 ± 0.86 e	179.62 ± 5.45 c
	B × NE	35.57 ± 3.21 b	231.78 ± 24.97 c
	B × WE	15.58 ± 1.42 de	111.97 ± 19.40 c

±—Standard deviation; a, b, c, d, e—different lowercase letters indicate statistically significant differences between means (homogeneous groups; Tukey’s HSD test *p* < 0.05).

**Table 4:** PM and CO emissions expressed per unit of fuel mass as affected by pellet species, electrostatic precipitator application, and their interaction.

Source of Variation	Item	PM (g Mg^−1^)	PM (g Mg^−1^ d.m.)
	Willow (W)	747.90 ± 365.53 a	795.04 ± 388.55 a
	Poplar (P)	391.21 ± 148.29 b	415.92 ± 157.23 b
Species (S)	Black locust (BL)	454.34 ± 148.15 b	483.08 ± 157.50 b
	Pine (PI)	383.90 ± 161.61 b	410.07 ± 172.51 b
	Birch (B)	444.17 ± 194.48 b	474.76 ± 207.18 b
Electrostatic precipitator (E)	Without (NE)	663.50 ± 227.73 a	706.61 ± 241.32 a
	With (WE)	305.11 ± 66.78 b	324.94 ± 70.42 b
	W × NE	1079.91 ± 41.15 a	1148.00 ± 43.41 a
	W × WE	415.88 ± 40.28 cd	442.09 ± 42.66 cd
	P × NE	513.91 ± 93.46 bc	546.36 ± 97.99 bc
	P × WE	268.51 ± 32.74 de	285.49 ± 34.05 de
S × E	BL × NE	585.71 ± 55.49 b	622.76 ± 58.92 b
	BL × WE	322.96 ± 3.67 de	343.40 ± 4.00 de
	PI × NE	520.17 ± 96.76 bc	555.61 ± 102.95 bc
	PI × WE	247.64 ± 15.24 e	264.53 ± 16.07 e
	B × NE	617.81 ± 58.91 b	660.33 ± 57.96 b
	B × WE	270.53 ± 25.21 de	289.18 ± 25.28 de

±—Standard deviation; a, b, c, d, e—different lowercase letters indicate statistically significant differences between means (homogeneous groups; Tukey’s HSD test *p* < 0.05).

**Table 5:** PM and CO emissions expressed per unit of fuel mass as affected by pellet species, electrostatic precipitator application, and their interaction.

Source of Variation	Item	CO (g Mg^−1^)	CO (g Mg^−1^ d.m.)
	Willow (W)	16,162.63 ± 1380.86 b	17,181.73 ± 1469.33 b
	Poplar (P)	30,556.78 ± 2614.73 a	32,492.28 ± 2724.46 a
Species (S)	Black locust (BL)	16,118.55 ± 2419.22 b	17,138.22 ± 2572.05 b
	Pine (PI)	3104.16 ± 898.73 c	3315.60 ± 958.67 c
	Birch (B)	2985.70 ± 1195.23 c	3190.56 ± 1271.52 c
Electrostatic precipitator (E)	Without (NE)	14,326.09 ± 10,907.52	15,238.80 ± 11,585.25
	With (WE)	13,245.04 ± 10,516.45	14,088.56 ± 11,177.44
	W × NE	15,390.59 ± 1623.57 b	16,360.46 ± 1719.42 b
	W × WE	16,934.67 ± 585.54 b	18,003.00 ± 646.14 b
	P × NE	31,444.39 ± 3792.24 a	33,433.23 ± 3945.07 a
	P × WE	29,669.17 ± 589.40 a	31,551.32 ± 580.45 a
S × E	BL × NE	17,795.09 ± 1379.79 b	18,920.90 ± 1467.52 b
	BL × WE	14,442.01 ± 2072.53 b	15,355.54 ± 2202.51 b
	PI × NE	2974.48 ± 1400.02 c	3176.71 ± 1493.39 c
	PI × WE	3233.84 ± 93.67 c	3454.50 ± 97.66 c
	B × NE	4025.91 ± 451.50 c	4302.69 ± 451.76 c
	B × WE	1945.49 ± 348.51 c	2078.43 ± 356.76 c

±—Standard deviation; a, b, c—different lowercase letters indicate statistically significant differences between means (homogeneous groups; Tukey’s HSD test *p* < 0.05).

**Table 6:** Selected statistical analysis indicators for the studied features (N Valid = 30).

Feature	Mean	Median	Min	Max	Q1	Q3	SD	CV (%)
PM (mg m^−3^) *	39.68	37.77	16.12	92.33	23.35	46.55	20.74	52.26
CO (mg m^−3^) *	1187.98	1338.71	119.06	2972.54	234.88	1580.90	933.87	78.61
η (%)	91.07	91.06	89.03	93.28	90.32	91.65	1.13	1.24
PM (mg MJ^−1^)	27.81	24.56	12.76	64.55	16.83	34.59	14.26	51.25
CO (mg MJ^−1^)	795.04	881.37	85.70	2066.67	184.63	1011.19	609.33	76.64
PM (g Mg^−1^)	484.30	426.04	230.07	1112.52	291.14	594.37	245.78	50.75
CO (g Mg^−1^)	13,785.56	15,205.05	1544.67	35,820.16	3318.10	17,509.93	10,541.78	76.47
PM (g Mg^−1^ d.m.)	515.77	453.07	246.00	1183.84	311.01	633.93	261.11	50.63
CO (g Mg^−1^ d.m.)	14,663.68	16,147.65	1651.64	37,986.23	3543.36	18,628.89	11,200.48	76.38

*—Air pollutant concentration normalized to standard conditions (101,325 Pa, 273.15 K) and to a reference oxygen content of 10% O_2_ in flue gas.

**Table 7:** Composition and properties of pellets. Own elaboration based on the results reported by Stachowicz and Stolarski [14].

Attribute	Willow	Poplar	Black Locust	Pine	Birch
Moisture (%)	5.93 ± 0.15	5.97 ± 0.24	5.95 ± 0.03	6.39 ± 0.08	6.48 ± 0.76
Volatile matter (% d.m.)	78.24 ± 0.04	77.79 ± 0.17	77.61 ± 0.28	80.12 ± 0.06	80.56 ± 0.19
Fixed carbon (% d.m.)	20.18 ± 0.07	20.44 ± 0.14	20.55 ± 0.32	19.69 ± 0.07	19.04 ± 0.21
Ash (% d.m.)	1.58 ± 0.06	1.78 ± 0.06	1.85 ± 0.13	0.19 ± 0.01	0.40 ± 0.03
Carbon (% d.m.)	53.36 ± 0.25	54.56 ± 1.06	53.18 ± 0.10	58.40 ± 0.25	58.37 ± 0.47
Hydrogen (% d.m.)	6.02 ± 0.09	6.11 ± 0.12	6.22 ± 0.08	6.48 ± 0.04	6.70 ± 0.06
Oxygen (% d.m.)	38.50 ± 0.34	37.00 ± 1.26	37.70 ± 0.14	34.82 ± 0.21	34.30 ± 0.53
Nitrogen (% d.m.)	0.50 ± 0.02	0.51 ± 0.07	1.00 ± 0.12	0.09 ± 0.00	0.20 ± 0.01
Sulfur (% d.m.)	0.026 ± 0.001	0.024 ± 0.001	0.035 ± 0.003	0.007 ± 0.000	0.012 ± 0.001
Chlorine (% d.m.)	0.020 ± 0.002	0.019 ± 0.003	0.021 ± 0.002	0.015 ± 0.001	0.015 ± 0.004
Higher heating value (HHV) (MJ kg^−1^ d.m.)	19.76 ± 0.03	19.86 ± 0.07	19.75 ± 0.07	20.73 ± 0.03	20.11 ± 0.09
Lower heating value (LHV) (MJ kg^−1^)	17.29 ± 0.05	17.36 ± 0.02	17.23 ± 0.04	18.00 ± 0.03	17.36 ± 0.13
Length (mm)	20.23 ± 0.69	16.57 ± 0.56	13.62 ± 0.46	12.40 ± 0.42	15.15 ± 0.52
Bulk density (kg m^−3^)	668.94 ± 1.73	663.56 ± 17.71	675.94 ± 2.67	648.33 ± 3.82	720.28 ± 10.49
Mechanical durability (%)	98.88 ± 0.11	98.27 ± 0.29	97.24 ± 0.23	99.06 ± 0.00	98.87 ± 0.04
Fines content (%)	1.12 ± 0.11	1.73 ± 0.29	2.76 ± 0.23	0.94 ± 0.00	1.13 ± 0.04

±—Standard deviation.

## Data Availability

The original contributions presented in this study are included in the article. Further inquiries can be directed to the corresponding author(s).
